# Dual Regulation of the *lin-14* Target mRNA by the *lin-4* miRNA

**DOI:** 10.1371/journal.pone.0075475

**Published:** 2013-09-13

**Authors:** Zhen Shi, Gabriel Hayes, Gary Ruvkun

**Affiliations:** 1 Department of Molecular Biology, Massachusetts General Hospital, Boston, Massachusetts, United States of America; 2 Department of Genetics, Harvard Medical School, Boston, Massachusetts, United States of America; 3 Biological Sciences in Dental Medicine Program, Harvard Medical School, Boston, Massachusetts, United States of America; Telethon Institute of Genetics and Medicine, Italy

## Abstract

microRNAs (miRNAs) are ∼22 nt regulatory RNAs that in animals typically bind with partial complementarity to sequences in the 3′ untranslated (UTR) regions of target mRNAs, to induce a decrease in the production of the encoded protein. The relative contributions of translational inhibition of intact mRNAs and degradation of mRNAs caused by binding of the miRNA vary; for many genetically validated miRNA targets, translational repression has been implicated, whereas some analyses of other miRNA targets have revealed only modest translational repression and more significant mRNA destabilization. In *Caenorhabditis elegans*, the *lin-4* miRNA accumulates during early larval development, binds to target elements in the *lin-14* mRNA, and causes a sharp decrease in the abundance of LIN-14 protein. Here, we monitor the dynamics of *lin-14* mRNA and protein as well as *lin-4* miRNA levels in finely staged animals during early larval development. We find complex regulation of *lin-14*, with the abundance *of lin-14* mRNA initially modestly declining followed by fluctuation but little further decline of *lin-14* mRNA levels accompanied by continuing and more dramatic decline in LIN-14 protein abundance. We show that the translational inhibition of *lin-14* is dependent on binding of the *lin-4* miRNA to multiple *lin-4* complementary sites in the *lin-14* 3′UTR. Our results point to the importance of translational inhibition in silencing of *lin-14* by the *lin-4* miRNA.

## Introduction

MicroRNA (miRNAs) are ∼22-nucleotide noncoding RNAs present in plant and metazoan phylogeny that repress gene expression post-transcriptionally. In animals, miRNAs usually repress target mRNAs by base-pairing to their 3′UTR, which have perfect or near-perfect sequence complementarity to nucleotides 2–7 (the “seed region”) of miRNAs, with bulges and mismatches in other regions of the miRNA-mRNA duplex [1]. The mechanisms by which animal miRNAs repress target mRNA stability or translation are still an open question. In particular, the relative contributions of mRNA degradation and translational repression in miRNA-mediated silencing are variable depending on the biological and experimental context (for review, see [2] and [3]). There are examples of miRNAs that primarily inhibit protein synthesis of their target mRNAs without affecting mRNA stability [4], [5], [6]. On the other hand, some analyses of other miRNA target genes only show a modest decrease in protein synthesis rate, arguing that most of the silencing is attributable to mRNA destabilization by miRNAs [7]. Although seemingly controversial, the multiple mechanisms of miRNA-mediated silencing are not necessarily mutually exclusive. Several recent studies monitoring the temporal effects of miRNA-mediated silencing gave rise to the hypothesis that miRNAs silence their target genes by an initial phase of translational repression, followed by mRNA deadenylation and decay, which further consolidate the silencing effect [8], [9], [10], [11], [12]. Even though this hypothesis reveals a dynamic process and reconciles some of the previous controversies, it should be noted that many of the studies were *in vitro* assays or experiments using synthetic reporters and/or via manipulating miRNA abundance. Moreover, the conclusions of the animal miRNA field have been plagued by the difficulty in assigning bona fide target mRNAs to miRNAs; as a result, the modest responses of hundreds of candidate miRNA targets may not represent the core mechanism that is subject to evolutionary selection. Therefore, instead of monitoring the entire genome, we focus on the *C. elegans lin-4* miRNA regulation of *lin-14*, an interaction that is biologically significant and subject to natural selection. *lin-14*, encoding a transcription factor, is a major target of the *lin-4* miRNA and contains seven conserved *lin-4* complementary sites in the 3′UTR [13], [14], [15]. Deletion of these complementary sites in *lin-14* gain-of-function mutants or a loss-of-function mutation in the *lin-4* miRNA disrupts the normal down-regulation of LIN-14 protein, which normally starts at the mid-first larval (L1) stage, and results in retarded heterochronic phenotypes [14], [16], [17].

Here, we monitored the abundance of *lin-14* mRNA and protein as well as *lin-4* miRNA levels in finely staged animals over the course of early larval development in both wild-type animals and mutants in which *lin-14* is relieved from repression by the *lin-4* miRNA. Our results revealed complex regulation of *lin-14*. In wild-type animals, *lin-14* mRNA levels first decline as soon as *lin-4* miRNA levels rise during the early L1 stage, accompanied by subsequent decrease in the abundance of LIN-14 protein. As *lin-4* miRNA levels rise during the late L1 stage, *lin-14* mRNA levels fluctuate with no obvious overall decline, whereas LIN-14 protein levels continue to decrease. In contrast, no decrease in the LIN-14 protein levels was observed in *lin-14* mutant animals in which the *lin-4*-complementary sites in the 3′UTR are deleted. Together, our analyses point to two phases of regulation: a fast *lin-14* mRNA destabilization phase, and long-term translational inhibition that is important in maintaining the silencing of *lin-14* by *lin-4* miRNA.

## Experimental Procedures

### Characterization of the *lin-14(n355)* inversion

RNA samples derived from wild type and *n355* were analyzed by 3′ RACE (Roche). The most prominent band that was detected in *n355* and not wild type corresponded to the length of the *lin-14* 3′UTR in the *n355* mutant that was deduced by Wightman et al. (1991). Sequencing of this band showed that at position 254 of the 3′UTR the *lin-14* sequence was fused to an intergenic region of the X chromosome corresponding to cosmid ZC373. PCR using primers homologous to *lin-14* and ZC373 and DNA from the *n355* mutant as template yielded bands that confirmed the chromosomal structure in the *n355* mutant that was predicted by 3′ RACE (data not shown).

### Quantitative Western blot

Odyssey CLx Infrared Fluorescent Western Blot was performed following the vendor's protocol. Briefly, NuPAGE® LDS sample buffer (2×) equal to the total volume of the worms was added and samples were boiled for 5 minutes. The lysates were pelleted in a microfuge and the supernatant loaded to a 4–12% Bis-Tris gel (Invitrogen) for electrophoresis. The proteins were transferred to nitrocellulose membrane (Bio-Rad). The membrane was blocked in blocking buffer (5% nonfat milk in PBS+0.1% Tween® 20) for 1.5 h at room temperature. Subsequently, the membrane was incubated in rabbit anti-LIN-14 (1∶1000) and mouse anti-actin (1∶5000, Abcam) primary antibodies at 4°C overnight. After washing four times with PBS+0.1% Tween® 20, the membrane was incubated in RDye 800 CW Goat anti-Rabbit IgG (1∶10000) and IRDye 680 RD Goat anti-Mouse IgG (1∶5000) at room temperature for 1 hour. The membrane was washed four times with PBS+0.1% Tween® 20, briefly rinsed with PBS and imaged with the Odyssey CLx infrared imaging system. LIN-14 and actin blots were scanned using the 800 nm and 700 nm channels, respectively. The abundance of LIN-14 was quantified by normalization to actin.

### Quantitative RT-PCR analysis of *lin-14* mRNA

Total RNA was purified using TRIzol Reagent (Molecular Research Center), DNase treated (TURBO™ DNase, Ambion), and reverse-transcribed using oligo(dT)_20_ primer (Invitrogen). The PCR amplification was monitored by SYBR green incorporation, and a corresponding threshold cycle (C_T_) was obtained. The quantity of *lin-14* mRNA, relative to *rpl-32*, was calculated using the formula 2^-ΔCT^, where ΔCT = (C_T_ *_lin-14_* – C_T_ *_rpl-32_*). The *lin-14* level was then normalized to its level at 0 h after release from L1 diapause. Primers used: *lin-14* forward primer spanning the last exon-exon junction (caaaaactgagagcgaaacg) and *lin-14* reverse primer in the last exon (tggaccttgaagaggaggag).

### Taqman miRNA Assays

Taqman miRNA Assays (Applied Biosystems) were performed following the vendor's protocol. Briefly, 100 ng total RNA was reverse transcribed using a miRNA-specific RT primer. The real-time PCR amplification was performed and a corresponding threshold cycle (C_T_) was obtained. The quantity of *lin-4* miRNA, relative to U6 as an internal reference gene, was calculated using the formula 2^-ΔCT^, where ΔCT = (C_T_ *_lin-4_* – C_T__U6_). The *lin-4* level was then normalized to its level at 0 h after release from L1 diapause.

### Northern Blot

We collected synchronized embryos and L4 staged animals of *lin-14(n536n540)/szT1* heterozygotes. *szT1* is a (I;X) translocation balancer chromosome that balances the slow growing and very small brood size of the *lin-14(n536n540)* homozygotes. While the *lin-14(n536n540)/szT1* mutants segregate homozygous larval lethal *szT1* homozygotes and homozygous *lin-14(n536n540)* animals, they constitute a minor component of the population because of the much larger brood and faster growth of the *lin-14(n536n540)/szT1* heterozygotes. Total RNA was purified using guanidium isothiocyanate disruption and purification through a CsCl cushion via ultracentrifugation, polyA selected, and separated on a 1.2% formaldehyde agarose gel. RNA was transferred to Nylon membrane, UV crossed linked, then hybridized to radiolabeled DNA probes that were generated from the 3.8 kb EcoRI fragment bearing the last 7 exons of *lin-14* and its 3′UTR. A DNA probe for the histone gene was used for normalization of mRNA content per lane, and a probe for the vitellogenin gene was used to indicate animal stages. X-Omat films exposed to the Northern blot were scanned on an optical scanner.

## Results

### Characterization of the *lin-14(n355gf)* mutant

**Figure 1:**
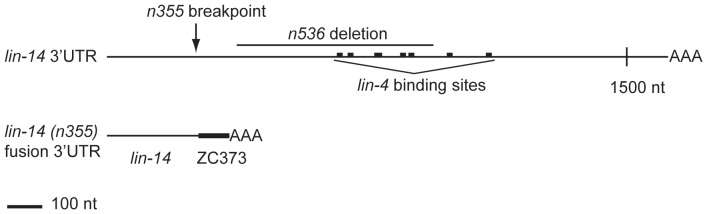
Diagram of the *lin-14* 3′UTR. Shown are the positions of the seven *lin-4* binding sites, which are all absent in the *n355* mutant allele. In the *n355* mutant, the 3′ end of the *lin-14* gene is fused to intergenic sequence corresponding to cosmid ZC373. The *n536* allele bears a 607-bp deletion in the *lin-14* 3′UTR, which deletes five of the seven *lin-4* binding sites.

The *lin-14(n355)* gain-of-function mutation was initially described as a possible translocation that separates the *lin-14* promoter and coding region from most of its 3′UTR, relieving it from repression by the *lin-4* miRNA [17]. To characterize the structure of the 3′UTR in the *n355* mutant we performed 3′ RACE (see Materials and Methods for details). We found that *n355* is an inversion that causes a break at nucleotide 254 of the *lin-14* 3′UTR and a fusion to an intergenic sequence of the X chromosome corresponding to cosmid ZC373, downstream of the gene *col-176* (the sequences flanking the fusion were *lin-14* to ZC373: *attatcccca*TCATTTCGAG). A series of adenosine residues that did not correspond to the genomic sequence, presumably indicating the polyadenosine tail, began at position 82 of the sequence derived from ZC373, suggesting that the *lin-14(n355)* 3′UTR is ∼335 nt, far shorter than the normally 1.6 kb wild-type *lin-14* 3′UTR (data not shown). The *n355* inversion removes all of the characterized complementary sites for *lin-4* [14] from the *lin-14* 3′UTR (Fig. 1). The *n355* mutation causes retarded heterochronic phenotypes and continued expression of the LIN-14 protein at larval stages later than the L1 stage, similar to a *lin-4* null mutant, further indicating that the *n355* mutation prevents the *lin-14* 3′UTR from responding to *lin-4* [13], [16].

### Temporal analyses revealed two phases of regulation of *lin-14* by *lin-4* miRNA

To resolve the dynamics of *lin-4* mediated down-regulation of *lin-14*, we performed temporal analyses of *lin-14* mRNA and protein as well as *lin-4* miRNA levels in finely staged wild-type and *lin-14(n355n679)* mutant animals collected at 3-hour intervals from the early first larval (L1) to early second larval (L2) stage at 20°C. The *n355* allele decouples *lin-14* from *lin-4* repression resulting in gain-of-function retarded phenotypes and slower development rate, but the *lin-14*(*n679)* V299D missense mutation confers a temperature-sensitive suppression of the *lin-14(n355)* retarded phenotype, due to a reduction-in-function mutation in the encoded LIN-14 protein. Therefore, while the LIN-14 protein stays at a high level after the L1 stage, that temporally misexpressed LIN-14 protein is non-functional for specification of L1 cell fates [18]. At 20°C, the *lin-14(n355n679)* mutant animals grow and develop at a similar rate to wild-type animals, allowing us to compare side-by-side the *lin-4* and *lin-14* dynamics in wild-type and *lin-14(n355n679)* mutant animals in which *lin-14* is relieved from repression by *lin-4*. More importantly, the misexpression of LIN-14 protein is decoupled from the mis-specification of L1 stage cell fates, so that molecular phenotypes can be interpreted without the complication of changes in developmental fate phenotypes.

**Figure 2:**
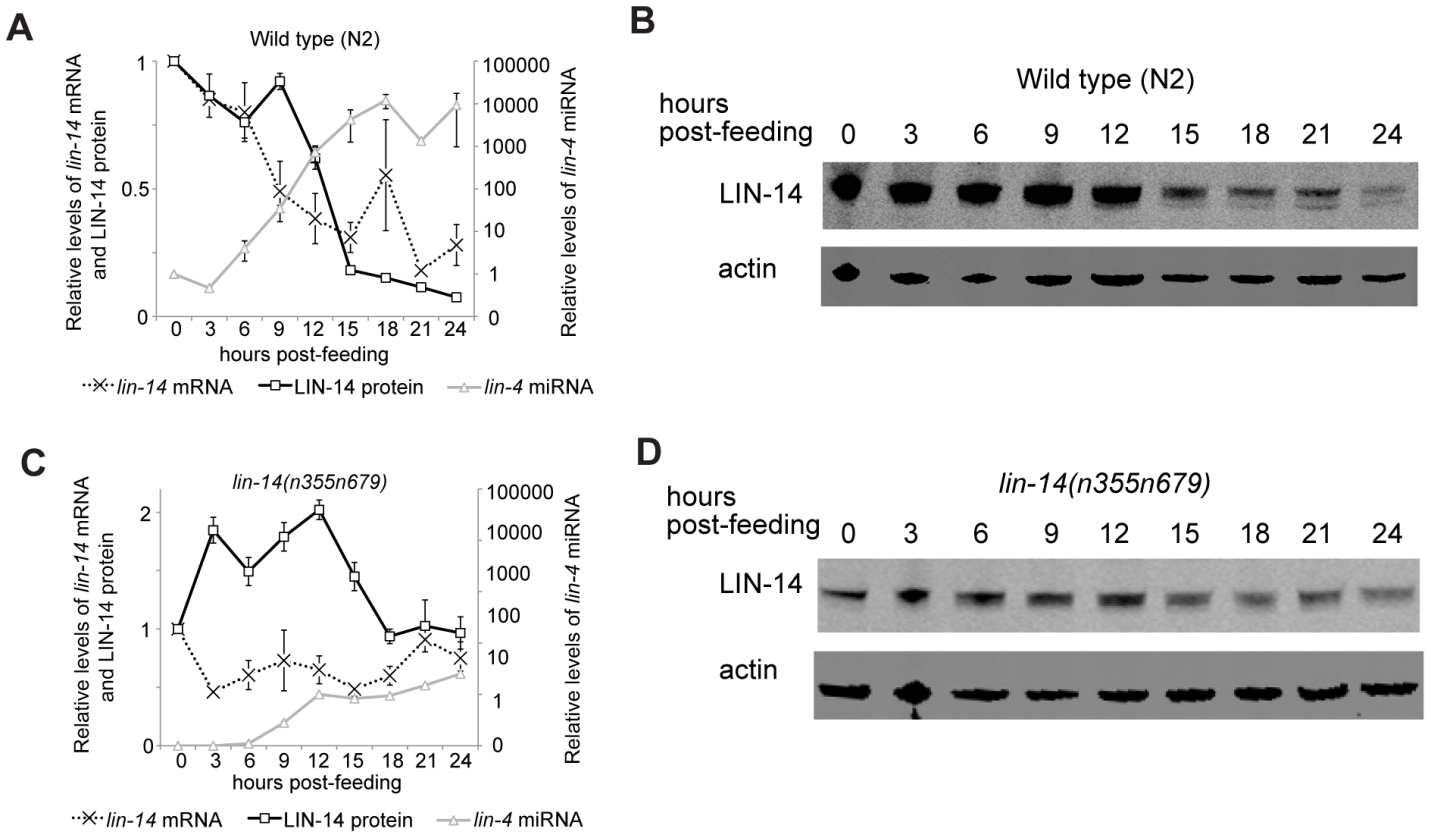
Temporal analyses of *lin-14* mRNA, protein, and *lin-4* miRNA levels in wild-type and *lin-14(n355n679)* mutant animals. (A, C) Quantification of *lin-14* mRNA, LIN-14 protein and *lin-4* miRNA from early L1 (0 hours post-feeding) to early L2 (24 hours post-feeding) in wild-type (A) and *lin-14(n355n679)* mutant animals (C). All results are shown relative to the level at the 0 hour time point. Error bars represent SEM for two independent experiments. (B, D) Representative immunoblots showing the abundance of LIN-14 protein in wild-type (B) and *lin-14(n355n679)* mutant animals (D) over 24 hours of development. Actin serves as a control for the normalization of LIN-14.

To quantitatively assay the full-length polyadenylated *lin-14* mRNA, we used oligo(dT)_20_ primer to reverse-transcribe mRNA isolated from synchronized animals and performed quantitative PCR (qPCR) analysis. Relative *lin-14* levels were obtained by normalization to *rpl-32*, a large ribosomal subunit that is abundant and stably expressed throughout development. The results are shown relative to the *lin-14* mRNA level at release from L1 diapause, or 0 hours of larval development. In wild-type animals, *lin-14* mRNA decreased to 49% and then to 38% of the 0 hour sample at 9 hours and 12 hours of larval development, respectively (Fig. 2A). From 12 hours to 24 hours of larval development, *lin-14* mRNA levels fluctuated with little evidence of further decline comparing the 24 hour to the 12 hour time point (p = 0.23, one-tailed t-test) (Fig. 2A). On the other hand, *lin-14* mRNA levels in the *lin-14(n355n679)* mutants fluctuated modestly, and showed little evidence of the monotonic reduction throughout the L1 stage as seen for wild type (Fig. 2C).

We monitored LIN-14 protein levels in the same samples that were analyzed for *lin-14* mRNA abundance. LIN-14 protein levels were analyzed by Odyssey CLx Infrared Fluorescent Western Blot and quantified by normalization to actin. The results are shown relative to the LIN-14 protein level at release from L1 diapause, or 0 hours of larval development. In wild-type animals, LIN-14 protein levels were stable during the first 9 hours of larval development (Fig. 2A and 2B). Subsequently, they declined to 62% and further to 18% at 12 hours and 15 hours (Fig. 2A and 2B), which followed the decline of *lin-14* mRNA levels that took place from 6–12 hours (Fig. 2A). Since LIN-14 protein levels declineed by ∼5 fold while *lin-14* mRNA levels declined by ∼2.5 fold, this suggests that both mRNA decay and inhibition of protein translation contribute to silencing of *lin-14* at early stages. From 15 hours to 24 hours of larval development, LIN-14 protein levels decreased from 18% to 8% of the 0 hour levels (Fig. 2A and 2B). In contrast, LIN-14 protein levels in the *lin-14(n355n679)* mutants did not show any significant down-regulation throughout the first larval stage (Fig. 2C and 2D), suggesting the ∼10-fold down-regulation of LIN-14 protein in wild-type animals is indeed mediated by *lin-4* miRNA binding to *lin-14* 3′UTR.

To track the mature *lin-4* miRNA levels, we performed Taqman miRNA assays in the same samples as above. In wild-type animals, *lin-4* miRNA is present at very low level in embryos, and can be detected by deep-sequencing [19] but not by Northern Blot, until 9–12 hours of larval development at 20°C [20], [21]. Consistent with previous studies, our analyses showed that *lin-4* miRNA levels began to rise substantially at 9 hours, and its level became ∼5000 fold higher at 24 hours compared to 0 hours. Curiously, the initial decline of *lin-14* mRNA levels between 6–12 hours happened prior to the significant accumulation of *lin-4* miRNA. When *lin-4* miRNA became abundant at later stages, *lin-14* mRNA levels fluctuated yet showed little evidence of significant down-regulation; however, LIN-14 protein levels continued to decrease. Together, our analyses point to two phases of regulation: a fast *lin-14* mRNA destabilization as soon as *lin-4* miRNA emerges, and long-term translational inhibition that initiates and is particularly important for maintaining the silencing of *lin-14* mRNA by the *lin-4* miRNA.

Strikingly, we found that *lin-4* miRNA levels in the *lin-14(n355n679)* mutants were ∼50–200-fold lower compared to stage-matched wild-type animals. We note that the mutant LIN-14 V299D protein encoded by the *lin-14(n355n679)* locus has reduced function. Thus the failure to up-regulate the *lin-4* miRNA in this *lin-14* mutant suggests that LIN-14 gene activity may normally act upstream of *lin-4* miRNA expression, which then feeds back on production of LIN-14 protein. The defect in LIN-14 protein activity in a *lin-14(n355n679)* mutant may break this autoregulatory loop. Alternatively, in *C. elegans*, miRNAs are protected from degradation by their target mRNAs [22], [23]. Therefore, it is possible that *lin-4* miRNA loses this protection in *lin-14(n355n679)* mutants due to the absence of *lin-4* binding sites in the *lin-14* 3′UTR. This would suggest that in contrast to models of hundreds of mRNA targets of miRNAs, in the case of *lin-4*, there may be just the *lin-14* mRNA target that is key for accumulation of the *lin-4* miRNA.

### The *lin-14* mRNA levels of wild-type and a mutant bearing a *lin-14* 3′UTR deletion are equal both at early and at late stages of animal development

**Figure 3:**
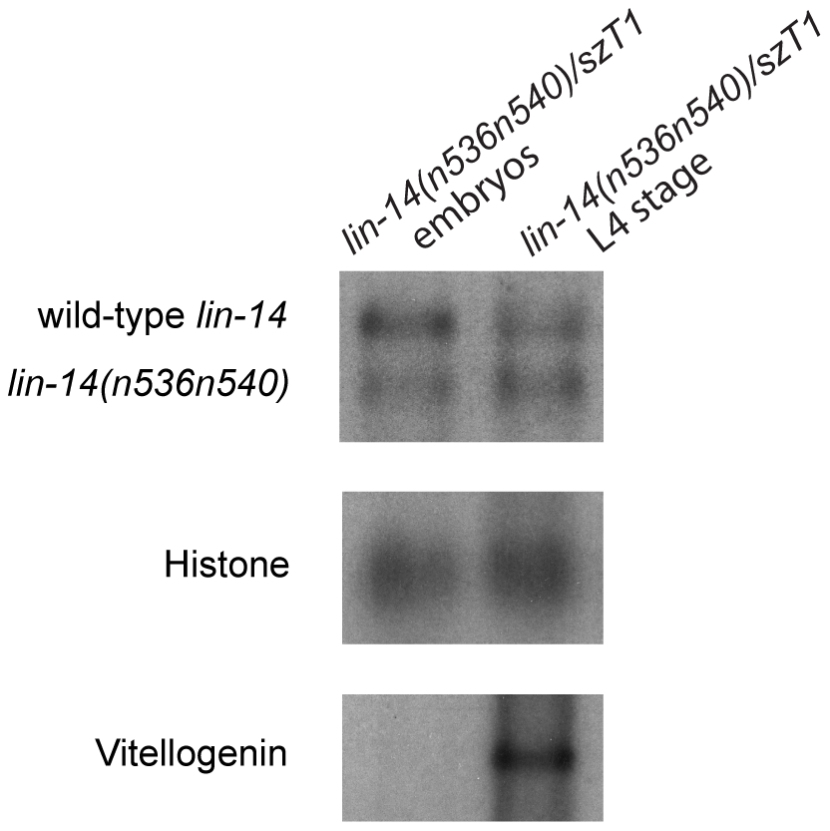
The levels of *lin-14* mRNA derived from the wild-type and the *lin-14(n536n540)* mutant allele bearing a 3′UTR deletion are similar. Shown are Northern blots of embryos and L4 staged *lin-14(n536n540)/szT1* heterozygotes, using probes from the *lin-14* (top), histone (middle) and vitellogenin genes (bottom). The levels of 3.5-kb wild-type *lin-14* and 2.9-kb *lin-14(n536n540)* mRNA are almost equal at both the embryonic and L4 stages. Histone mRNA is used as a control for even loading. Vitellogenin mRNA, encoding yolk polypeptides, which is most abundant during oogenesis, is shown to indicate animal stages.

Another approach to assay whether *lin-4* repression acts via mRNA destabilization or translational inhibition is to compare the levels of wild-type *lin-14* mRNA to mutant *lin-14* mRNA missing *lin-4* complementary sites, in a heterozygous animal. To this end, we assayed *lin-14* mRNA levels in the *lin-14(n536n540)/szT1* heterozygous strain with one wild-type and one *n536n540* allele of *lin-14*. *lin-14(n536)* bears a 607-bp deletion that removes five of the seven *lin-4*-complementary elements in the *lin-14* 3′UTR (Fig. 1) and is a gain-of-function mutation causing retarded heterochronic development due to derepression of *lin-14* [14], [17]. The *n540* allele, an amber mutation at position 280 (Lys to amber), was isolated as a recessive suppressor of the *lin-14(n536gf)* mutant [16], [24]. We reasoned that if *lin-4* miRNA silences *lin-14* by enhancing mRNA decay, then *lin-14(n536n540)* mRNA should be stabilized relative to the wild-type *lin-14* mRNA due to reduction of *lin-4*-mediated regulation. However, Northern blot of RNA isolated from *lin-14(n536n540)/szT1* heterozygotes showed that the 3.5-kb wild-type *lin-14* and 2.9-kb *lin-14(n536n540)* mRNA levels are similar at both the embryonic stage, before *lin-4* miRNA is expressed, and at the L4 stage, after *lin-4* repression has occurred (Fig. 3). This experiment indicates that there is very modest down-regulation of *lin-14* mRNA between the pre-*lin-4* expression stage and the L4 stage from either the wild-type allele or the *lin-14* allele missing the majority of the *lin-4*-complementary regions. However, the two residual *lin-4* complementary sites in the *lin-14(n536)* 3′UTR are insufficient to mediate the silencing effect by *lin-4* through translational repression, because the *n536* mutation derepresses expression of the LIN-14 protein at the L2 and later stages. Our result is also largely consistent with a recent study [25] and further stresses the importance of translational inhibition in *lin-4* miRNA-mediated silencing of *lin-14*.

## Discussion

The results we present here consolidate the earlier studies where *lin-14* was found to be silenced by *lin-4* without being significantly destabilized at the mRNA level [4], [14], as well as a recent ribosome profiling study showing translational control of *lin-14* by *lin-4* [25]. Although cases have been reported where regulation by miRNAs can cause destabilization of target mRNAs in *C. elegans* [26], the relative contributions of mRNA degradation and translational repression in miRNA-mediated repression are variable [5]. For example, *lin-41* mRNA levels regulated by the *let-7* miRNA showed the strongest reduction, whereas *lin-14* mRNA showed modest reduction [26]. However, the *lin-41* mRNA degradation noted by Bagga et al. [26] was not observed by Stadler et al., who instead suggested translational control of *lin-41* by the *let-7* miRNA [25].

Several recent studies have suggested a two-phase model for miRNA-mediated silencing that begins with translational repression, followed by mRNA deadenylation and decay, which consolidates the silencing [2], [8], [9], [10], [11], [12]. Interestingly, we observed distinct dynamics for *lin-4* silencing of *lin-14*. First, *lin-14* mRNA level declines to ∼40% as soon as *lin-4* miRNA is first expressed during 6–9 hours of L1 development, prior to the decrease of LIN-14 protein level. Although translational inhibition may also contribute to this initial silencing of *lin-14*, currently this hypothesis is not supported by solid evidence. On the other hand, between 12–24 hours of larval development, *lin-14* mRNA levels fluctuate with no obvious further decline, whereas LIN-14 protein levels continue to decrease. Furthermore, by the early L2 stage, the LIN-14 protein level has declined by more than 10 fold and the *lin-14* mRNA level has declined by ∼3 fold, suggesting that long-term translational inhibition maintains silencing of *lin-14* mRNA by the *lin-4* miRNA.

It remains unclear why the modes of action of miRNA-mediated silencing are different depending on the biological and experimental context. In particular, it is intriguing why and how the predominant mechanism of silencing could change even for the same miRNA and its target mRNA in the same organism. Since the levels of *lin-4* miRNA increase more than 5000 fold from the early L1 stage, when *lin-14* mRNA just begins to decay, to the early L2 stage, when *lin-14* has been stably silenced, we hypothesize that the number of *lin-4* molecules binding to the *lin-14* mRNA 3′UTR might determine the mode of silencing. Specifically, it is possible that a single or a few *lin-4* miRNA molecules binding to *lin-14* 3′UTR can cause mRNA decay, whereas more miRNAs binding causes translational control. Because there are seven *lin-4* complementary sites in the *lin-14* 3′UTR, this would trigger a fast decline in LIN-14 protein synthesis even when the level of *lin-4* is low, to achieve a sharp transition in development. To maintain silencing, translation of *lin-14* mRNA is inhibited probably through binding of multiple *lin-4* molecules to the *lin-14* mRNA 3′UTR as *lin-4* massively accumulates. Supporting this hypothesis, we found that the *lin-14(n536n540)* mRNA missing five out of the seven *lin-4*-complementary elements in its 3′UTR is destabilized to a similar level as the wild-type *lin-14* mRNA. However, *lin-14(n536)* is not properly silenced and causes a retarded heterochronic phenotype [14], [17]. This suggests that the two remaining *lin-4* complementary sites might be sufficient to cause the normal *lin-4*- triggered decay of *lin-14* mRNA, but insufficient to induce *lin-4*-triggered translational repression.

Finally, our fine-stage analysis showed that *lin-14* mRNA drops initially during the first larval intermolt stage, but returns to a higher level during the L1-L2 molt. It is an emerging trend that mRNA levels could oscillate in animals entering and exiting the molt. Therefore, an important lesson is that looking at a single time point could be misleading, which may contribute to the differences in results and conclusions obtained from different labs.
